# “Dietaly”: practical issues for the nutritional management of CKD patients in Italy

**DOI:** 10.1186/s12882-016-0296-5

**Published:** 2016-07-29

**Authors:** Claudia D’Alessandro, Giorgina Barbara Piccoli, Patrizia Calella, Giuliano Brunori, Franca Pasticci, Maria Francesca Egidi, Irene Capizzi, Vincenzo Bellizzi, Adamasco Cupisti

**Affiliations:** 1 Department of Clinical and Experimental Medicine, University of Pisa, via Roma 67, 56126 Pisa, Italy; 2 Department of Clinical and Biological Sciences, University of Turin, Turin, Italy; 3 Néphrologie, Centre hospitaler Le Mans, Le Mans, France; 4 Department of Movement and Wellness Sciences, University of Naples Parthenope, Naples, Italy; 5 Nephrology and Dialysis Unit, Trento Hospital, Trento, Italy; 6 Nephrology and Dialysis Unit, USL Umbria 1, Perugia, Italy; 7 Division of Nephrology Dialysis & Transplantation, University Hospital “San Giovanni di Dio e Ruggi d’Aragona”, Salerno, Italy

**Keywords:** CKD, Low-protein diet, Dietary sodium, Dietary phosphorus, Simplified diet, Nutrition, Dietary treatment renal insufficiency

## Abstract

Evidence exists that nutritional therapy induces favorable metabolic changes, prevents signs and symptoms of renal insufficiency, and is able to delay the need of dialysis. Currently, the main concern of the renal diets has turned from the efficacy to the feasibility in the daily clinical practice.

Herewith we describe some different dietary approaches, developed in Italy in the last decades and applied in the actual clinical practice for the nutritional management of CKD patients.

A step-wise approach or simplified dietary regimens are usually prescribed while taking into account not only the residual renal function and progression rate but also socio-economic, psychological and functional aspects.

The application of the principles of the Mediterranean diet that covers the recommended daily allowances for nutrients and protein (0.8 g/Kg/day) exert a favorable effect at least in the early stages of CKD. Low protein (0.6 g/kg/day) regimens that include vegan diet and very low-protein (0.3-0.4 g/Kg/day) diet supplemented with essential amino acids and ketoacids, represent more opportunities that should be tailored on the single patient’s needs.

Rather than a structured dietary plan, a list of basic recommendations to improve compliance with a low-sodium diet in CKD may allow patients to reach the desired salt target in the daily eating.

Another approach consists of low protein diets as part of an integrated menu, in which patients can choose the “diet” that best suits their preferences and clinical needs.

Lastly, in order to allow efficacy and safety, the importance of monitoring and follow up of a proper nutritional treatment in CKD patients is emphasized.

## The mainstays of a general dietary approach to CKD

The modification of quantity and quality of dietary proteins are the mainstay of nutritional therapy in patients affected by chronic kidney disease (CKD). The Recommended Daily Allowance (RDA) represents the intake of nutrient that is safe for 95 % of the general population: for proteins, the RDA value is 0.8-1.0 g/Kg/day. Hence low protein regimens are featured by a dietary protein intake < 0.8 g/Kg/day.

In a recent international consensus it has been confirmed that protein restriction is the core issue of dietary manipulation in CKD patients [1]. It includes a “low-protein” (0.6-0.7 g/Kg/day) diet up to a” very low protein” (0.4-0.3 g/Kg/day) diet supplemented with essential amino acids and ketoacids. However, protein restriction is only a part, thought a very relevant part, of a more complex dietary management of CKD patients [2].

Phosphate intake should be reduced (700–400 mg/day) as well as sodium intake (2–3 g/d). Dietary energy intake must cover energy requirement up to 35 Kcal/Kg/d for patients < 65 years and 30 Kcal/Kg/day for patients > 65 years old. Qualitative aspects of foods (plant or animal origin, fresh or preserved, cooking modality, …) are important, as well. The main characteristics of the dietary therapy in CKD are resumed in Table 1.

**Table 1:** Main general features of the dietary therapy in patients with chronic renal insufficiency

Energy	30–35 Kcal/kg/day
Protein	0.3–0.7 g/kg/day
Carbohydrates	At least 60 % of total energy intake
Lipids	At least 30 % of total energy intake
Sodium	2.3 g/day (equivalent to 6 g NaCl salt)
Phosphate	<700 mg/day
Supplements	Essential amino acid and ketoacids, calcium carbonate, vitamins, iron

It is largely proven that nutritional therapy induces favorable metabolic changes, prevents signs and symptoms of renal insufficiency, and is able to delay the need for dialysis [3–5]. Currently, the main concern of the renal diets has turned from efficacy to the feasibility in the daily clinical practice [6].

Although various practical strategies should be implemented, some general rules must be considered and accompany dietary protein manipulation. They include nutrients, special foods and supplements.

Energy and protein intake are closely related. When protein restriction is needed, high energy intake is mandatory to spare protein from being used as energy substrate, and allow a normal metabolic adaptation to a low protein intake [7, 8]. This prevents the loss of lean muscle mass and the onset of protein-energy wasting. For this reason special attention should be directed toward over-weight/obese CKD patients in which a negative energy balance must be avoided, when protein intake is less than 0.8 g/Kg/day. Instead, when loss of body weight is needed, we recommend a protein intake of 0.8-1.0 g/Kg/day.

During low-protein regimens, energy intake is guaranteed by the use of grains and fats, preferably of vegetable origin (extra-virgin olive oil is probably the best one). The use of protein-free products represents a valuable tool to give energy mainly via carbohydrates, that are almost free of nitrogen, phosphorus, potassium and with an amount of sodium often less than the corresponding regular food [9].

Below, more information will be given regarding protein-free products and food selection.

By far, the classical energy recommendations are based upon a series of elegant, although relatively old studies, that are still considered appropriate for reference and that suggest an energy intake of at least 30 Kcal/Kg/day for people over 60 years old and above 35 Kcal/Kg/day for younger patients. However, some evidence suggests that these targets may be much more than enough, especially in sedentary patients [10].

The control of dietary phosphorus intake is crucial from the early stages of CKD [11]. Several aspects should be considered to reduce the net intestinal absorption of phosphorus and the tendency toward a positive phosphate balance [12, 13]. Some foods such as dairy, yolk and nuts have a very high content of phosphorus and therefore should be avoided or rarely consumed. Phosphorus is present in foods rich in proteins, thus a reduction of the protein intake limits the phosphorus intake. Within the same food group, there are foods defined as “more favorable” than others, where “favorable” means less phosphorus for the same protein content. A phosphorus to protein ratio of 12 mg/g can be considered the “watershed” for identification of most favorable foods [13]. However, food selection may not be enough because some foods contain phosphate-based preservatives [14, 15], that are highly absorbable phosphates (over 90 %). Counseling to identify and avoid foods with phosphorus-based preservatives is essential, as is the need to pay more attention to nutrition facts labels on packaging. Conversely phosphate is present in plants as phytate (a form of storage). As humans cannot split phytate, the net intestinal absorption from this source is quite poor. However, the content of phosphorus in nuts is very high, 400 mg per 100 g as average, so the effective phosphorus load remains significant even supposing a 30-40 % bioavailability.

A simple, home-employed procedure to reduce the effective content of phosphorus, other than sodium and potassium, is to boil the food [13].

**Fig. 1:**
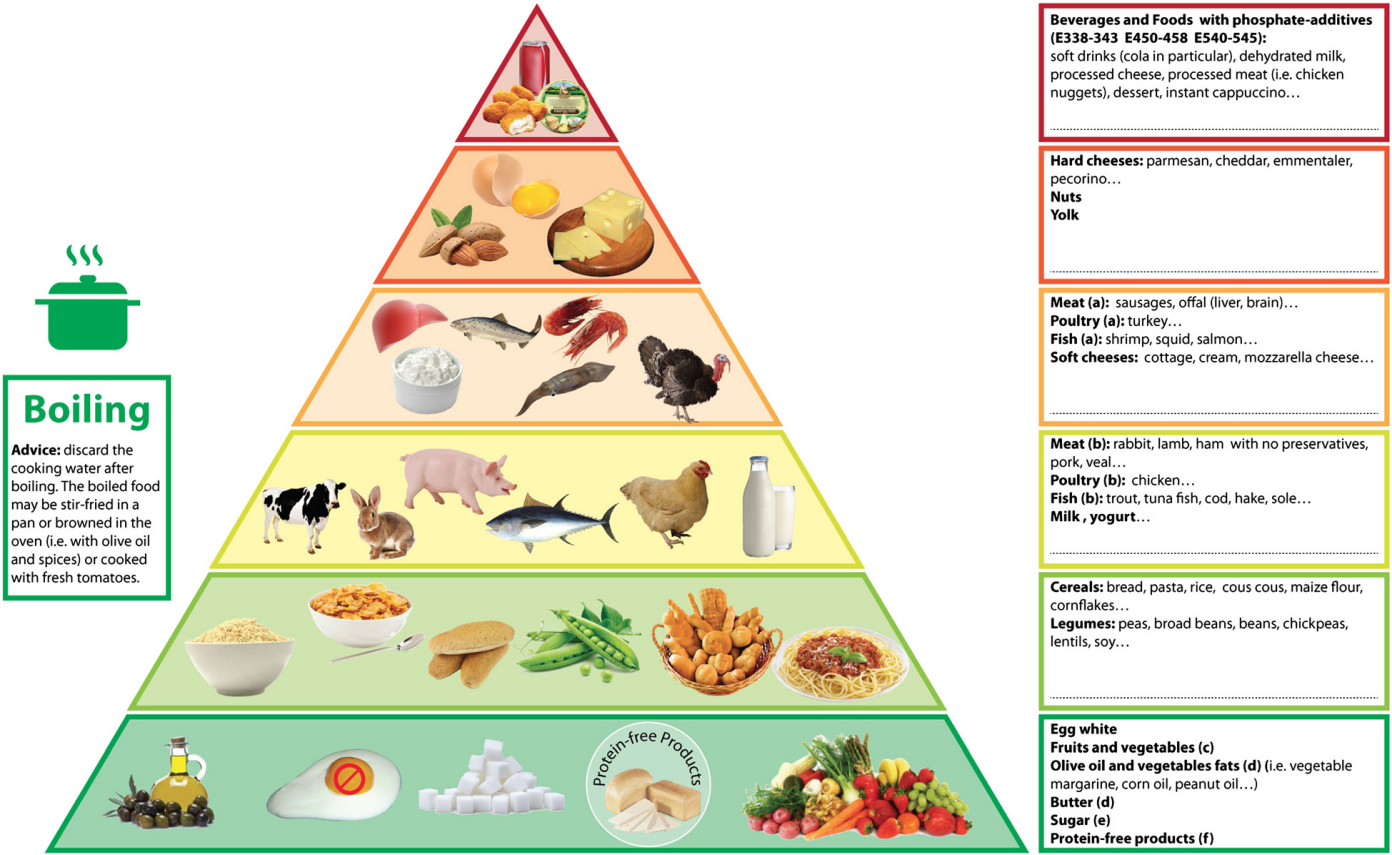
The Phosphorus Pyramid (with permission, ref. no. 16)

The use of educational tools such as brochures with images can be useful in summarizing the essential aspects of dietary phosphate control in CKD patients. “The phosphorus pyramid” (Fig. 1) represents a simple, immediate and even fun tool to summarize the main points for reducing the effective phosphorus loading [16]. The pyramid has been designed as a guide for Italian health professionals and patients, and represents the food choices with the most frequently used Mediterranean ingredients. However it can be adapted to different countries and habits.

The distribution of food on the various floors should support the choice without the need to memorize the phosphorus content of each food item. The pyramid consists of six floors in which foods are arranged on the basis of the phosphorus content, phosphorus to protein ratio and phosphorus bioavailability. Each level has a colored edge (from green to red) that corresponds to recommended intake frequency. The boxes on the right provide further information and are intended to be tailored to the individual patient: namely, specific foods may be added (in the empty spaces in the boxes on the right) during counseling, based on phosphorus content, phosphorus to protein ratio, and bioavailability.

The boiling pot on the left side suggests boiling as the best cooking method to reduce the phosphorus content. To improve taste and appearance, the box provides the suggestion that boiled food can be simmered with olive oil, garlic and parsley, browned in the oven with olive oil and spices, or cooked with fresh tomatoes.

As an initial general approach, the suggested intake of sodium is 2–3 g/day equivalent to 6 g of sodium chloride (NaCl) salt, which is equal to the WHO recommendation: Stricter sodium restrictions may be prescribed in patients with moderate to severe sodium-water retention. However, evidence exist that very low sodium intake may be associated to unfavourable outcome [17].

It is well known that salt intake reduction is important to limit the tendency towards water retention and to enhance the effect of drugs used to correct hypertension and proteinuria and in general for nephro- and cardiovascular protection.

For this reason, counseling should discourage the use of processed foods (meats and cheeses in particular) and precooked meals (these usually contain NaCl salt as a preservative and flavor enhancer) and encourage the consumption of fresh food. For example we suggest to avoid adding salt in the water for pasta cooking and to add a pinch of salt (on the basis of nephrologist’s indications) to the seasoning sauce. We also suggest not adding sauce to the pasta until the sauce is well seasoned or to use herbs and spices (pepper, basil, thyme …) to make the food more attractive and palatable. In all cases the suggestions may be tailored to patients’ habits.

In the early CKD stages, with the exception of interstitial diseases in which water and sodium waste are common, sodium restriction may be the most important dietary intervention [17]. In more advanced CKD stages there are concerns for loss of concentration ability of the failing kidney. In such a setting the combination of ACE inhibitors or angiotensin II receptor blockers, diuretics and sodium restriction may be dangerous, especially in summer and in cases of fever. Hence, in our country where summers may be hot, we often discontinue or soften sodium restriction, especially in patients with severe CKD. In many Italian regions bread is an important source of salt (1.6 g/100 g), hence we suggest “Tuscan bread”, that has a low content of salt (0,18 g/100 g). Tomato sauces ready to use are another source of salt (0.7 g/100 g): so we suggest fresh tomatoes or “simple” canned tomatoes (salt content: 0.02 g/100 g) as the preferred choice.

**Fig. 2:**
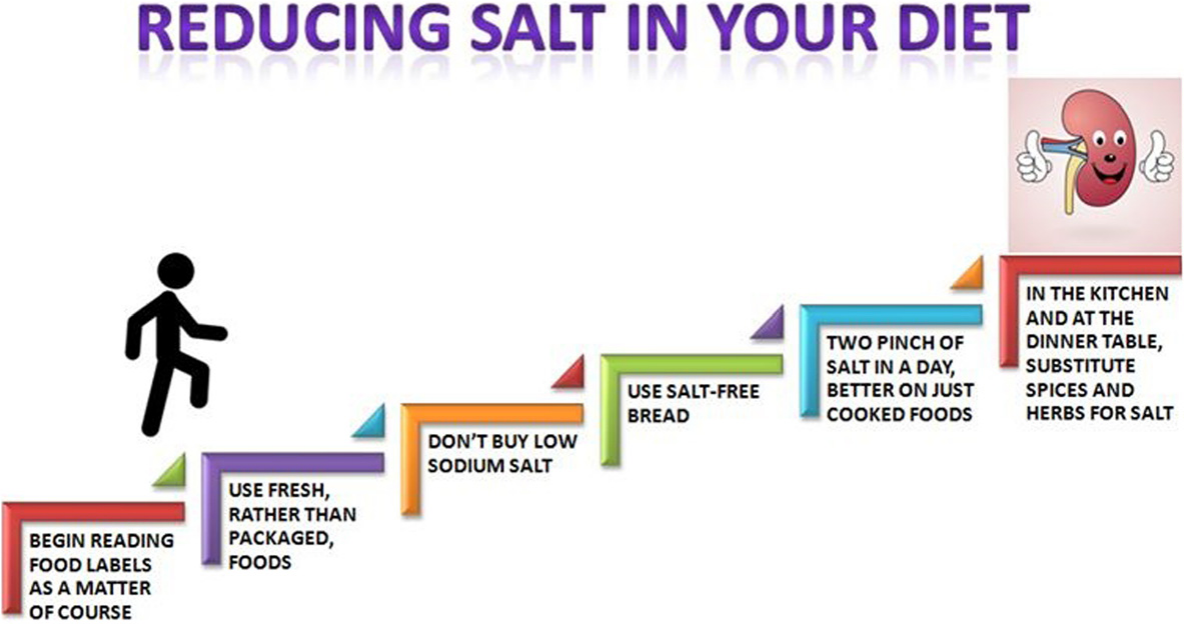
Step by step visual tool to reduce salt intake

Simple images could help patients to retain the most important messages given during the counseling and to remember them when at home. Here we show a simple visual tool, with a step by step approach, that we used to help patients to reduce salt consumption (Fig. 2).

Elevated potassium intake may contribute to hyperkalemia. It is a real risk only in more advanced CKD stages, in hemodialysis patients, or during therapy with one or more renin-angiotensin system inhibitor drugs. Hyperkalemia is often linked to or worsened by metabolic acidosis, because of the shift of potassium in the vascular compartment. This is one more motivation for a full correction of metabolic acidosis in CKD patients. Bicarbonate is a sodium salt, that must also be considered during sodium restricted regimens.

Some practical dietary advice to limit the risk of hyperkalemia is listed below.

- Avoid fruit rich in potassium such as bananas, kiwi, apricots, grapes, smoothies, natural fruit juices, particularly if concentrated. For example an orange juice could be prepared using only one orange and then diluted with water. The orange juice counts as a fruit serving.
- Avoid nuts (e.g. walnuts, hazelnuts, peanuts, etc.), seeds (e.g. Sunflower seeds, pumpkin seeds, etc.) and dried fruits (e.g. dried plums or dates).
- Fruit consumption is allowed as follows: no more than two serving a day (one serving = 150 g) of apples, pears, strawberries, fresh plums; or one serving a day of oranges, tangerines; or half a serving a day of cherries, pineapple, peach.
- Legumes, as other plant-origin foods, are rich in potassium and should be boiled in plenty of water and cooking water should be discarded. Legumes are widely used in the Italian cuisine: they are a source of protein of good biological value and should be used together with grains as a substitute for meat, fish etc. In the Vegan diet they should be eaten daily.
- Prefer boiled vegetables: boiling is the best cooking method to remove minerals. Once boiled vegetables can be seasoned and flavored in various ways: stir-fried in a pan with flavor, in the oven, added to tomato sauce (in Italy tomato sauce is highly used). The same should be applied to potatoes: peeling before boiling reduces potassium content. The cooking water should always be discarded. It is preferable to avoid steaming or directly cooking in the oven or microwave.

It is likely that boiling induce loss of water soluble vitamins, as well. This is a reason why water soluble vitamin preparations may be supplemented in CKD patients.

It is noteworthy that in CKD patients on protein restricted diets, it is mandatory to guarantee high energy intake, adequate essential amino acids supply and correction of metabolic acidosis. This are the conditions needed to obtain a correct adaptation to protein restriction and to avoid a negative nitrogen balance [18].

To this aim, important tools for targeting energy and amino acids adequacy are represented by the protein-free products as well as the mixtures of essential amino acids and ketoacids [19].

### Protein-free products

Protein-free products are a very important tool for the safe and successful implementation of an animal-based low-protein diet or a very low protein diet for CKD patients. They represent a source of energy from carbohydrates free from nitrogen, and with a low to negligible content of potassium, sodium and phosphorus [9]. Energy/phosphate ratio is particularly favourable using these products (Table 2).

**Table 2:** Energy to phosphate ratio (Kcal/mg) in protein-free products and in regular foods

Food	Energy/phosphate (Kcal/mg)	
	Regular food	Free/low-protein products
Pasta	1.88	8.5
Bread	3.57	6.6
Biscuits	2.64	22.4
Rusks	3.87	4.93
Milk	0.53	29.1

Protein-free products have been conceived to supply energy while limiting the nitrogen load of uremic patients when dialysis could not be offered.

In the 60s, in England, wheat starch began to be used to produce starch-made derivatives of cereals such as bread, pasta, biscuits, etc. In 1966 the first protein-free pasta was produced in Italy. In 1977 flour, biscuits, and crispbread became available. More recently, protein-free drinks (a milk substitute), snacks, new kinds and sizes of bread became commercially available. The organoleptic characteristics of these products have been greatly improved over time, but poor palatability and limited choices still represent a barrier to a widespread use of protein-free foods. They are less palatable than corresponding regular products due to different reasons [20–23].

In the last few years the food industry has tried to create alternative production routes to obtain products similar to the regular ones with quite good results [24, 25]. In protein-free bread, the fiber content is significantly higher than in regular bread because the added fibers allow the rising of the dough. In the last year more fibers have been added to low protein bread and pasta also with the aim to reduce the glycemic index of these products. Unfortunately they cannot recreate the fragrance and the typical color of the crust because they are the result of the reaction of proteins and carbohydrates with heat (Maillard reaction).

The role of renal care professionals becomes relevant in advising and training CKD patients for achieving an optimal preparation and use of protein-free products

### Supplements

A mixture of essential amino acids (EAA) and Ketoacids (KA) have been used to supplement VLPD in patients with advanced CKD. Most EAAs are at first degraded via transamination, which involves removal of the amino group bounded to the α carbon and its replacement by a keto or hydroxyl group. The KA can be degraded further by oxidation. KA or hydroxyl acid (HA) analogues of the EAAs, except for lysine and threonine, can be transaminated to form the respective EAAs. This process uses circulating amino groups, and prevents their incorporation into urea or other toxic nitrogenous waste products.

Transamination of KA or HA to synthesize EAA would occur only if significant amounts of the KA or HA analogues are available and a deficit of the corresponding EAA exists. Conversely, with a surfeit of the EAA the enzyme kinetics would appear to favour degradation of KA or HA rather than formation of new EAA [26].

The available mixtures of KA and HA contain four KAs (of the EAAs valine, leucine, isoleucine, and phenylalanine), the HA analogue of methionine and four EAAs (thryptophan, histidine, threonine, and lysine). KA and HA analogues are strong acids: for this reason KAs are given as a salt, generally a calcium salt. The commercially available products are branded as Ketosteril®, which is distributed all over the world, and Alfa-Kappa® that is present only in Italy; the two products are similar except for the absence of Thryptophan in Alfa-Kappa® tablets.

Initially, it was thought that substantial quantities of urea are hydrolyzed in patients with CKD and the amino groups released from urea could aminate KA analogues. Then, studies showed that only small amounts of urea can be reused for amino acid synthesis, so the reduction in net urea generation with LPD or supplemented VLPD is due more to decreased amino acids degradation and reduced urea synthesis than to reuse of amino groups released from hydrolyzed urea [27–29].

KA and EAA supplements contain substantial amount of the KA of leucine, which may suppress protein degradation. In contrast, leucine may increase protein synthesis in muscle: hence both leucine and ketoleucine and also KA and EAA supplements may promote net protein anabolism and suppress protein catabolism [30]. In a rat model, animals fed a sVLPD with KA and EAA show evidence of suppression of apoptotic and ubiquitin pathways in muscles and an increased protein synthesis and inhibited protein degradation [31].

In patients treated with KA and EAA supplementation, an increase in serum calcium levels and a decrease in serum phosphate levels generally occur. This is probably due to the very low phosphorus content of VLPD and to the calcium supply from KA supplements. Each tablet contains 43 mg calcium that must be taken into account when total calcium intake is to be defined [32]. The reduction of acid generation by low protein intake and by the plant origin foods included in the diet contribute to prevent or correct metabolic acidosis. It can improve protein balance and slow progression of CKD [33]. Another important effect reported in patients treated with supplemented VLPD is a reduction of serum PTH levels with improvement in bone histology [34].

Because of the important reduction of milk and dairy intake to limit salt and phosphorus load, CKD patients may need a supplementation of 400–800 mg elemental calcium. Usually calcium is given as carbonate and so it may contribute to counteract metabolic acidosis. It is noteworthy that one tablet of Ketosteril® or Alfa-kappa® contain 43 mg of elemental calcium.

Patients on a supplemented VLPD regimen, in order to meet the need of vitamin B, require a daily (or every two days) supplement of a complex of vitamins B (usually B1, B6, B12), and a daily dose of folic acid. Vitamin B12 is mandatory on the vegan diet while rarely it is necessary a vegan diet is alternated with the low protein diet that provides a daily amount of meat or fish containing vitamin B12. Also iron supplements are given in patients with CKD in particular in those who follow plant-based renal diets.

## Dietary and nutritional approaches for CKD patients: practical applications

Based on the above mentioned general dietary issues in CKD patients, we describe different dietary approaches developed and practised in Italy in the last decades for the nutritional management of CKD. Hence the following kinds of diets reflect the Italian habits and culture, but we hope that several kinds of diets and dishes can be applied and implemented in other countries, as well.

### A “step-wise” approach

The method of the nutritional intervention for CKD patients is not only related to the residual renal function and its progression rate but depends also on the socio-economic, psychological and functional status of the patient. Hence, a careful evaluation of dietary habits represents the first step of any nutritional intervention and gives the qualitative and quantitative information needed to develop a targeted counseling. In this regard dietary recall (3-day food recall, food journal, etc.) is an essential tool that help the dietitian (but also nephrologists and nurses) to estimate habitual energy and nutrient intake and to better understand patient’s life-style. In Appendix 1 you will find a simple tool to assess dietary habits in daily practice. It’s a brief questionnaire that is not a real food frequency questionnaire but it could be very useful to obtain important information for dietary counseling in a little time (Appendix 1).

There are four main dietary regimens that can be applicable in CKD patients (Table 3). It is noteworthy that in all of these cases, energy intake must always cover energy requirement; only when protein intake is greater or equal to 0.8 g/Kg/day, may a moderate low-energy diet, if needed, be implemented.

**Table 3:** Dietary regimens in CKD. The degree of protein and phosphorus restriction is mostly dependent on severity of renal insufficiency, whereas adequacy of energy intake and limitation in salt intake are generally prescribed. As body weight (bw) we refer to the ideal body weight, or to the adjusted weight (when applicable)

Dietary regimen	Main composition features	CKD stages
a) Healthy diet, according to WHO	Protein: 0.8 g/Kg bw/day according to RDA.	I, II, IIIa
	Sodium according to WHO recommendation (2.3 g/day); Phosphorus according to RDA (700 mg/day)	
b) Low Protein Diet (LPD)	Protein: 0.6 g/Kg bw/day, whose 0.4 g/Kg of animal origin; Sodium: 2.3 g/day; Phosphorus < 700 mg/day	IIIb, IV, V
c) Low Protein Vegan Diet	Protein : 0.7 g/Kg bw/die, from grains and legumes;	IIIb, IV
	Sodium : 2.3 g/day; Phosphorus : < 700 mg/day	
d) Supplemented Very Low Protein Diet (sVLPD)	Protein: 0.3-0.4 g/Kg bw/day supplemented with EAAs and KAs	IV, V
	Sodium: 2.3 g/day, Phosphorus : 300–400 mg/day	

#### Healthy diet, according to WHO recommendation

The “normalization” of protein intake to the RDA value of 0.8 g/kg/day, represents a protective measure for most of CKD patients as well as for the general population. Maintaining protein intake above this value cannot be justified as it may produce waste products whose retention depicts the biochemistry and clinical pattern of renal insufficiency. Moreover, “normalized” protein intake for most patients means a protein reduction in respect to their habits, especially in young/adult people who usually report a protein intake greater than 1.2 g/kg/day. On the opposite, elderly patients often have inadequate energy and nutrients intake so, for them, a normalization means to increase their usual protein (and energy) intake.

In this dietary regimen the patient is trained to follow the rules of a healthy diet so foods from the various groups (grains, meat, fish, beans, vegetables, milk and dairy products) may be consumed with the precautions described below. These are the reason why it is important to define the habitual food intake. This does not mean being bound to weight scales but it’s just a way to become visually familiar with the size of the serving. The use of domestic measures (Table 4) or reviewng the weight on package labels may help the patients to quickly define the appropriate measures.

**Table 4:**
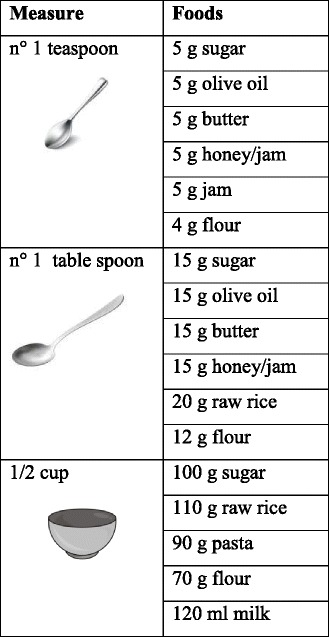
Kitchen measurements

- The use of protein-free products is not strictly required when protein intake is “normal”. However, when a higher energy intake is necessary (i.e. patients who do vigorous exercise) if we augment the serving size to reach the needed caloric intake we risk giving too much protein. In this case protein-free products represent a source of “green energy” for CKD patients

-To reduce restriction in the variety of food choice we can act on the frequency of consumption. For example dairy products have a high content of salt, phosphorus, saturated fats, so they are not a good choice for CKD patients, but the suggestion to avoid cheese is not well accepted. On the contrary, indication suggestions to eat cheese once a week or every ten day is welcome. The suggestion is not perceived as a prohibition and predisposes the patient to better follow the other suggestions.

### Practical issues for food selection

*Grains* (bread, pasta, rice, barley, corn etc.). They must always be present, preferably at every meal.

*Meat and Poultry*. There is not much difference regarding the protein content of “white” or “red” meat; it is better to limit these to 1–2 times a week in the amount defined by the dietitian. The consumption of processed meats (sausages, cold cuts) is not recommended because of the high salt content and the potential presence of phosphorus-based preservatives.

*Fish*. Everyone knows that fish is preferable to meat but only for the quality of fats. Patients often think they can eat fish in free amounts but, as regards proteins, they have to respect the recommended amount. It can be consumed 2–3 times a week.

*Beans* (beans, chickpeas, peas, lentils, etc.). They are a good source of protein of good biological value (not really complete as those of meat and fish) and should be used as a substitute for meat, fish etc., together with grains and not as a side dish. We suggest to consume beans several times a week.

*Dairy products*. They provide proteins, but have a high phosphorus content. Hard cheeses are also rich in salt so it is desirable to avoid them and limit the consumption of fresh cheese to once every 7–10 days. Dietitians will evaluate the exclusion of milk and yogurt, according to the patient’s dietary habits and clinic, and will give suggestions on how to replace it with rice, oats or almond drink and derivatives, if necessary. Another question is the use of Parmesan cheese: as just a small amount over pasta (i.e. two teaspoons) provides salt and phosphorus it is important to assess whether it is appropriate to include or avoid it.

*Egg*s: we recommend an occasional consumption of whole egg as the yolk is rich in phosphorus. On the contrary the egg white can be eaten more freely, as it contains proteins of high biological value and very little phosphorus.

*Oils and fats*. Olive oil is the best source of fats of plant origin and it contains oleic acid, a mono-saturated fat with anti-oxidant properties. Nephrologists and dietitians may allow the use of animal fats like butter or cream in those patients with poor appetite to improve food palatability and to increase the energy density of the meal.

*Vegetables*. Fruits and vegetables give variety to meals, are rich in fiber and vitamins as well as minerals such as potassium (for this reason their consumption might need some precautions).

#### Low protein (0.6 g/Kg/day) diet: when a Health, normal protein diet is not enough

The protein intake is reduced but proteins must be mostly of high biological value. Meanwhile energy intake must be adequate to spare protein from being used as a fuel. For this reason the introduction of protein-free products (their characteristic are described above) is recommended as they represent a source of energy without wasting products.

### Practical issues for food selection

*Grains*. They are replaced by protein-free products

*Meat and Poultry*. They have to be present every day and, along with fish, they represent the only source of proteins with high biological value. The daily amount is defined according to the body weight. The consumption of processed meats (sausages, cold cuts) is not recommended because of the high content of salt and possibly of phosphate (added as preservatives). The use of ham without polyphosphates is allowed, because, being cooked, it has a lower salt than salami, sausages etc..

*Fish*. For fish it is applied the same as for flesh. We ask to moderate the consumption of shellfish because of the high phosphorus content.

*Egg. W*e suggest the consumption of the egg white because it is rich in proteins of high biological value without significant phosphorus content.

*Oils and fats*. Olive oil is the best source of fats, as described above. Nephrologists and dietitians may allow the use of animal fats like butter or cream in those patients with poor appetite to improve food palatability and to increase energy density of the meal.

*Vegetables*. Fruits and vegetables give variety to meals, are rich in fibers, vitamins as well as minerals such as potassium, so, in some cases, their consumption needs the precautions already mentioned.

#### The “Vegan” low-protein (0.7 g/kg/day protein) diet: changing the quality of proteins and foods

This diet is a valid alternative to the standard low-protein diet with protein-free products, suitable for those who do not like these products or for those who are often away from home and finally for those who do not like animal products such as meat and fish (necessary as a source of proteins when consuming protein-free products) [35, 36].

In the vegan diet the consumption of grains and beans is mandatory. Grains are generally considered only as a source of carbohydrates and legumes are often the only vegetable used as a side dish to meat and fish. Actually they both have a high content of protein (e.g. 100 g of pasta has 10–11 g proteins on average and 100 g of dried legumes contain 22–23 g of proteins: for comparison 100 g of meat contains 20 g of protein on average), they are not “complete” as meat and fish but when consumed together (e.g. pasta and beans, pasta and chickpeas) provide an adequate protein intake both from the qualitative and quantitative point of view.

### Practical issues for food selection

*Grains*: (bread, pasta, rice, barley, corn etc.) must always be present, preferably in every meal.

*Beans*: (beans, chickpeas, peas, lentils, etc.) are a source of protein of good biological value but they are not complete as those of meat and fish because they lack methionine. They should be used as a substitute for meat, fish etc., together with grains. In the Vegan diet they should be eaten daily. The cooking method is very important: legumes should be boiled in plenty of water and the cooking water should be discarded. They should be eaten together with grains because they contain methionine so the combination of legumes and grains gives an adequate amount of essential amino acids both quantitatively and qualitatively.

*Oils and fats.* Olive oil is the best source of fats as described above. Nephrologists and dietitians may allow the use of animal fats like butter or cream in those patients with poor appetite to improve food palatability and to increase energy density of the meal.

*Vegetable*. Fruits and vegetables give variety to meals, are rich in fibers and vitamins as well as minerals such as potassium, so, in some cases, their consumption needs the precautions described above.

#### The very low protein (0.3-0.4 g/Kg/day) diet supplemented with amino acids and keto-acids: when a severe restriction is needed to postpone dialysis

Protein intake is further reduced and is not enough to provide essential amino-acids to cover the daily requirements so a supplementation with specific products, namely amino-acids and keto-acids is mandatory [19]. It is a vegetarian diet with protein-free products. A list of foods allowed or to avoid is given below (Table 5).

**Table 5:** Foods allowed and to avoid with sVLPD

Food included in the diet	Food excluded from the diet
• Protein free products: pasta, biscuits, bread, breadsticks • Grains (rice, corn, buckwheat) • Vegetables, raw or cooked • Fresh fruit or fruit in syrup^a^ • Jam, marmalade, sugar, honey^a^ • Olive oil, vegetable margarine • Cream, butter^b^ • Spices and herbs	• meat, fish, sausages and cold cuts • eggs • milk and dairy products • nuts^c^ • bread, breadsticks, biscuits, pasta and biscuits made with plain flour • meat extracts • soft drinks cola-based in particular

^a^To avoid in case of diabetes; ^b^to avoid in case of overweight/obesity, cardiovascular diseases; ^c^to avoid in case of hyperkaliemia

In terms of protein supply, this vegetarian diet without integration with EAAs and KA is inadequate. Hence the supplementation is mandatory to obtain the advantages that this dietary therapy can provide, in terms of nutritional adequacy. [1, 37, 38].

## The simplified tips diets

Major goals of this dietary regimens for CKD patients are adherence and safety. The idea is that compliance to low-protein diet is low and major determinants of adherence are patient’s understanding (knowledge) and satisfaction (achieving the objective, support) [39]. To pursue this aim requires skilled dietitians, individualized dietary programs, intensive educational programs, regular counseling [40] which required dedicated personnel, are time and money consuming [41] and are rarely available in the common clinical practice [42]. Moreover, it’s quite common that patients do not completely understand even the personalized dietary plans which are often complicated and sometimes quite distant from their actual dietary habits. Furthermore, young, active patients commonly do not eat lunch/dinner at home, but in restaurants or at work. As a result, these habits often reduce the adherence to low protein diets do not allow patients to achieve the protein targets and thus lead to low energy intake (skipped meals) [43]. An option to overcome this practical limitation is represented by simplified, very practical approaches which may be considered either when patients have several barriers to follow a standard LPD or when dietitians are not available.

An example of this simplified dietary approach for a low-salt diet, consists in a number of tips that impact the daily eating habits [44]. Dietary sodium restriction (100 mmol/day, that is 6 g/day of salt, sodium chloride) represents the primary intervention to achieve the blood pressure control in CKD due to its salt sensitivity. However, this intervention in CKD patients is very disappointingly since less than 20 % of patients adhere to this prescription. Rather than a structured dietary plan, a list of basic recommendations to improve compliance with a low-sodium diet in CKD may allow patients to reach the desired salt target in the daily eating:

1. Move the salt shaker away from the table
2. Cook pasta, rice, and cereals without salt (add in smaller amount directly on cooked food)
3. Use spices (e.g., herbs, lemon, vinegar, hot pepper) instead of salt or salt containing condiments (e.g., ketchup, mayonnaise, mustard, barbecue sauce)
4. Look for the amount of sodium on food labels
5. Choose fresh or plain frozen foods and low-salt bread
6. Cut back on frozen dinners, canned soups, packaged mixes, cured meat and fish (eg, ham, bacon, anchovies, salmon)
7. Choose fresh (e.g., mozzarella) rather than seasoned cheese
8. Rinse canned foods (e.g., tuna) to remove some sodium
9. Abolish salty snack foods (e.g., chips, nuts)

This approach has been demonstrated in the clinical practice to lower the salt intake in hypertensive, high-salt eater CKD patients, and to reduce the blood pressure values by lowering of the extracellular volume expansion.

A further example of a simplified tips approach for a “conventional low-protein diet”, consists of 6 tips [45]. To help patients to adhere to dietary prescriptions it is crucial to offer them simple and attractive approaches to the diet. A simplified and easy dietary approach for the low-protein diet, consisting of 6 simple tips, was recently evaluated in a randomized clinical trial where this strategy was compared to a “normalized” low-normal protein (0.8 g/kg/day) diet. The decrease of protein and phosphate intakes were greater in patients following the 6 tips approach and almost 50 % more patients were adherent to the prescription (44 vs 70 %, respectively in 6-tips and LPD). This approach implies a further step in the scale of compliance that is the concordance among physician and patient and allows for very promising results. This simplified approach for LPD is particularly useful when dietitians are not available: patients are recommended to continue to eat according to their habit and preference, while introducing the following restrictions:

1. Do not add salt while cooking or eating
2. Avoid any kind of salami, sausages, cheese and dairy products or canned food
3. Replace noodle or bread with free-protein products
4. Consume the second course (meat, fish and eggs) only once a day
5. Consume 4–5 servings per day of fruits or vegetables
6. Once a week the main course may be a “normal” noodle with legumes instead of the second course, with fruit and vegetables. To note, in Italy there is the habit to divide the main course dish into two dishes, the first one based on pasta (plain preparation), and the second one based on meat or fish or cheese or eggs, with fresh vegetables on the side. This strategy could replace the more complex “standard low-protein diet” and improve both metabolic profile and adherence to the dietary plan with respect to a standard LPD in CKD patients.

## Simplified and moderately restricted low-protein diets

This approach sees low protein diets as an integrated menu, in which patients should chose the “diet” best suited to their preferences and clinical needs [46]. To improve compliance these suggestions should be followed:

1. Chose the diet you prefer (or that which bothers you less)
2. Change it if you want to try a different approach
3. Do not weight food (qualitative approach)
4. Enjoy 1–3 unrestricted meals per week.

To allow an easy shift among diets, the different diets were designed with a common frame, including a general part, regarding the diet “philosophy” and a variable one.

The four diets presently employed are identified according to the target protein intake expressed as g/Kg/day (0.6 with protein free food; 0.6 vegan supplemented; 0.6-0.8 vegan non supplemented; 0.3 vegan with protein free- products, supplemented with essential amino acid and ketoacids). Since food is not weighed, the control of protein intake is mainly based upon the assessment of urinary urea appearance [47]. See Appendix 2.

## Monitoring and Follow up: the way for the efficacy and the safety of the nutritional treatment

**Fig. 3:**
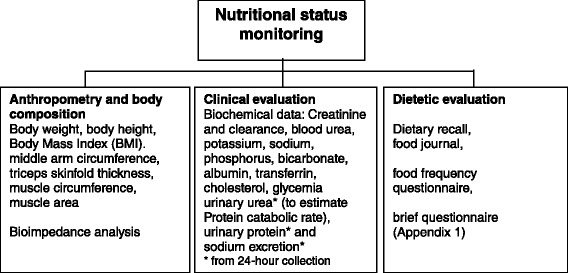
Tools for monitoring of nutritional status in CKD patients

Malnutrition is an important concern in patients with CKD and inadequate intakes of nutrients may play a very relevant role. Thus the assessment of nutritional status and nutrients intake and its regular monitoring is of extreme importance to detect signs of malnutrition as soon as possible. Here we present some assessment tools (Fig. 3) that could be used in daily clinical practice.

### Timing of the dietary-nutritional visits

The first control visit should be not later than 2–3 weeks from beginning of the nutritional therapy and the second should be one month later. Following visits should be planned every 3 or more months, unless otherwise clinically indicated.

There are two main and different approaches to this issue: if the dietary assessment is a part of the routine nephrological assessment, the number of visits is established upon the severity of the disease: for example, at least monthly in ND-CKD stage 5, at least every 2 months in stage 4 CKD and at least every 3–4 months in stage 3b. The frequency of controls is further modulated according to the presence and entity of proteinuria, comorbidities and of the type of disease (i.e. immunologic-systemic patients are usually seen more frequently). As a rule, patients on VLPD or at high risk of malnutrition should be controlled more frequently. If, on the contrary, the dietary approach is self-standing, once compliance and understanding are achieved, one control every 3–4 months may be more than enough in stable, compliant patients on moderately restricted diets; however, patients on VLPD need to be controlled more frequently.

The availability of a telephone number or e-mail address may be useful to solve questions or doubts about diet, to reinforce the counseling and to maintain a continuous link with the patients that, in turn, may strengthen the adhesion to dietary prescriptions.

It is noteworthy that this policy of “distant counseling” is not recognized by the Italian National Health Care System, so that it mainly relies on voluntary basis. Caregiver commitment is a tool for attaining patient commitment, and this point should be stressed in a period in which western doctors are in a difficult balance between being skilled technicians (replaceable by other skilled technicians) and clinical and personal references in the context of an holistic clinical approach (in other terms…. no committed patient without committed caregivers). Moreover, the epidemiology of CKD patients has changed in the last 20 years, in terms of age, diabetes and cardiovascular morbidity, leading to a more complex and various dietary management [48–50].

## Let’s go to practice: look at some dietary plans and some recipes!

We report below three dietary plans (Tables 6, 7 and 8), one for LPD, one for Vegan diet and one for VLPD and some recipes as simple examples of how to turn the suggestions given above.

**Table 6:** A day with… a low protein (0,6 g/kg) diet

Breakfast	
• 150 g rice drink or protein-free drink with a teaspoon of sugar • n° 5 protein-free crisp toasts • n° 2 spoons marmalade	
Mid-morning snack	
• Protein-free sandwiches with tomatoes or stir-fried vegetables (see the recipe below)	
Lunch	
• 100 protein-free spaghetti with olives • Fish (90 g) and vegetables salad (see the recipe below) • 50 g protein-free bread • 150 g apple	
Mid-afternoon snack	
• 30 g low protein crackers	
Dinner	
• 80 g protein-free pasta with broccoli (see the recipe below) • Strips of chicken (70 g) with sage and rosemary (see the recipe below) • Vegetables • 50 g protein-free bread • 150 g pear

**Table 7:** A day with…a Vegan diet

Breakfast
• 150 g rice drink or oat drink • 5 normal crisp toasts
Snack
• “Bruschetta”: bread (preferably toasted) with rubbed garlic, olive oil and tomatoes
Lunch
• Rice (80 g) with lentils (30 g dry lentils) (see the recipe below) • vegetables • 50 g low-salt bread • 150 g apple
Snack
• 100 g strawberry ice cream (see the recipe below)
Dinner
• Pasta (70 g) with chickpeas (20 g dry chickpeas) (see the recipes below) • vegetables • 50 g low-salt bread • 150 g pear

**Table 8:** A day with…a very low protein diet

Breakfast	
• 150 g rice drink or oat drink with a teaspoon of sugar • 6 regular crisp toasts • n° 3 spoons of marmelade/honey	
Snack	
• 30 g low protein crackers	
Lunch	
• Protein-free pasta (100 g) with olives (see the recipe below) • vegetables • 50 g protein-free bread • 150 g apple	
Snack	
• 100 g strawberry ice cream	
Dinner	
• rice (80 g) with lentils (see the recipe below) • vegetables • 50 g protein-free bread • 150 g pineapple	

The VLPD is not adequate in terms of essential amino acids intake. A supplementation with essential amino acids is absolutely needed, as described above in the text

The amount of each ingredients has to be changed on the basis of patient needs.

In these three examples the amount of the ingredients are calculated for an hypothetical subject 1.70 m high and a BMI = 23.5 Kg/m^2^.

What follows represents, when it is possible, an typical Italian meal structure. Both lunch and dinner include a first dish with pasta (in the case of low protein diets we used free-protein pasta) and a second course based on meat and fish in the case of the LPD, legumes in the case of the Vegan diet and vegetables in the case of the VLPD. The second course is generally served along with a side dish of vegetables raw o cooked (preferably boiled to control potassium intake, if necessary).

For people who work and are not able to have lunch at home we suggest a different meals structure: Lunch includes a main course with meat or fish, a source of protein that is easy to find in a restaurant or to brought from home. The dinner includes a first dish with protein free pasta since protein-free products cannot be found in a restaurants. Obviously patients can distribute the amount of the food provided in the dietary plan throughout the day as they wish and according to their needs.

## Low protein recipes

**Table:** 

*Low protein sandwiches*	Fig. 4
Ingredients: ✓ a slice of protein-free bread cut into 4 square pieces ✓ 50 g tomato ✓basil and/or origan ✓No 1 table spoon of olive oil	
**Notes:** These sandwiches can be eaten as a snack or appetizer. Tomato can be replaced by other types of vegetables, previously boiled, cut into small pieces and stir-fried with olive oil and spices. These sandwiches can be freely consumed when using protein-free bread unless the nephrologist or the dietitian give different indication on the amount (e.g. in the case of overweight / obesity) This recipe can be used with the low protein diet and with the very low protein diet.	
*Protein-free spaghetti with olives* Ingredients: ✓ 100 g protein-free spaghetti ✓ 100 g ripe tomatoes ✓ 20 g green olives ✓a pinch of red pepper ✓ 1/2 clove of garlic ✓ No 2 table spoons of fresh parsley, chopped ✓ No 2 table spoons of olive oil	Fig. 5
**Notes:** Chop the garlic and the parsley finely; crushes the olives and chop coarsely. Put everything in a pan with olive oil and pepper to taste. Cook slightly and add the peeled tomatoes. Crush with a fork. The dressing should be cooked for only 5 min. Boil the pasta in plenty of water and drain “al dente” and add it to the sauce in the pan. Mix everything well and serve.	
*Protein-free pasta with broccoli* ✓ 100 g protein-free pasta ✓ 100 g broccoli ✓ No1 garlic clove peeled and halved ✓ No 2 low protein breadsticks ✓ a pinch of pepper ✓ ¼ cup fresh parsley, chopped ✓ No 2 table spoons of olive oil	Fig. 6
**Notes:** Boil the broccoli in plenty of water, discard the cooking water. Stir fry the garlic in a spoon of oil add the broccoli and pepper. Meanwhile, in a food processor, combine breadsticks with a tablespoons of oil; pulse until coarse crumbs form. Boil the pasta in plenty of water and drain “al dente”. Reserve 1/2 cup of water to be used to cook pasta. Add the broccoli to pasta, mix well and then cover with breadcrumbs and parsley before serving.	
*Fish and vegetables salad* Ingredients: ✓ 90 g tilapia ✓ 80 g zucchini ✓ 30 g red onion ✓ 50 g yellow pepper ✓ 3 cherry tomatoes ✓ No 2 table spoons of vinegar ✓ No 2 table spoons of olive oil ✓ Plenty of parsley	Fig. 7
**Notes:** Cut the zucchini, the onion and the pepper into small wedges, boil them in plenty of water and vinegar, discard the cooking water. Boil the fish in another pot, drain well and add to the vegetables, add the cherry tomatoes cut into wedges, the parsley and season with olive oil.	
*Strips of chicken with sage and rosemary* Ingredients: ✓ 70 g chicken (breast) ✓ No 1 ½ table spoon of olive oil ✓ Sage and rosemary to taste	Fig. 8
**Notes:** Wash and chop the herbs and put them in a pan with a spoon of oil, heat up and add the strips of chicken. Be careful that the oil does not burn. Stir-fry quickly, join the cooking juice and serve.	

*Vegan recipes*

**Table:** 

*Rice with lentils* Ingredients: ✓ 80 g rice ✓ 40 g dry lentils ✓ No 1 table spoon of onion, cut into small slices ✓ A pinch of pepper (if you like) ✓ No 1 spoon of olive oil ✓ No 1 teaspoon of vegetable margarine ✓ Vegetable broth made with carrot, onion, tomato and celery	Fig. 9
**Notes:** Boil the lentils in plenty of water, avoid soaking and discard the cooking water. In a saucepan, sauté the onion in the oil add the lentils, pepper and vegetable broth and simmer over low heat. Boil the rice in another pot, drain well and add to the lentils, stir well and slowly adding a teaspoon of vegetable margarine.	
*Pasta and chickpeas* Ingredients: ✓ 80 g pasta ✓ 50 g potato ✓ 100 g chickpeas, previously boiled ✓ No 1 table spoon of onion cut into small slices ✓ No 1 table spoon of celery cut into small slices ✓ No 2 table spoons of olive oil ✓ a small ripe tomato ✓ 1/2 clove of garlic ✓ A small spring of rosemary ✓ A pinch of pepper (if you like)	Fig. 10
**Notes:** Peel the potato, cut into pieces and boil in water. Dice the tomato and put it into a pan with 1 tablespoon of olive oil. Add the onion, the celery, the garlic and the rosemary. Cover with water and cook as long as they are soft. Remove the rosemary and add 3/4 of the chickpeas. Cook a few more minutes and then blend it all with an immersion blender. Add the remaining chickpeas and add water as needed. Cook for fifteen minutes and then add the pasta. Finish cooking. Add a spoon of olive oil (and black pepper if you like) before serving.	
*Strawberry ice cream (without milk)* Ingredients (3–4 servings) : ✓ 300 g strawberry pulp ✓ 120 g sugar ✓ No 1 table spoon of lemon juice	Fig. 11
**Notes:** Put the strawberries in a blender with the lemon juice. Pour into a bowl, add sugar and mix well. Put in the freezer for one hour, then pass it to the mixer to break up the ice crystals and put in the freezer for two hours. This recipe can be used also with the low and very low protien diets.	

## Abbreviations

CKD, chronic kidney disease; EAA, essential amino acids; HA, hydroxyl acids; KA, Ketoacids; LPD, low protein diet; ND CKD, non-dialysis chronic kidney disease; RDA, recommended daily allowance; VLPD, very low protein diet

## Appendix 1

**Table 9:** Questions and tools for quickly assessing CKD patient’s dietary habits

Do you	Yes		Not	
1. Usually shop and prepare your own food?				
2. Read food nutrition labels?				
3. Think that a dietary treatment could be a therapy				
In an average week, how often do you:	Usually/Often	Sometimes	Rarely/Never	Does not apply to me
4. Skip breakfast?				
5. Eat a meal take out restaurants?				
6. Drink milk and yogurt?				
7. Put the cheese on your pasta?				
8. Eat beef, pork, chicken, turkey?				
9. Eat fish?				
10. Use regular processed meats?				
11. Use sauce as ketchup and mayonnaise?				
12. Eat high sodium processed foods like canned soup or frozen/packaged meals, chips?				
13. Add salt to foods during the cooking or at the table?				
14. Drinks soft drink like soda and cola?				
15. Eat nuts, peanuts, pistachio nuts				
16. Do less than 30 total minutes of physical activity 3 days a week or more?				
17. Eat pulse	dried	canned		frozen

## Appendix 2

**Table 10:** The diets presently employed are identified according to the target protein intake expressed as g/Kg/day (0.6 with protein free food; 0.6 vegan supplemented; 0.6-0.8 vegan non supplemented; 0.3 vegan with protein free- products, supplemented with essential amino acid and keto-acids)

0.6 with protein free food	0.6 vegan supplemented	0.6-0.8 vegan not supplemented
Our diet plans to replace bread and pasta-rice-flour with protein free-food; fruits and vegetables can be taken without restriction; meat, fish cheese, etc. can be used as “seasoning” for the pasta, or as part of the “main course” that has to be completed as much as possible with vegetables.	Our diet is based upon foods of plant origin only (fruit, vegetables, sugars, starches such as pasta and bread, cous-cous, polenta and legumes) supplemented by tablets of a mixture of amino acids and “ketoacids (Alfa-Kappa® or Ketosteril®). These tablets allow you choosing the type of plant derived food that you prefer without needing to integrate the different types of vegetable proteins (grains and legumes at each meals). This is a simplified diet, but do not forget the pills… The supplements are calculated as 1 each 8–10 Kg of body weight; their amount depends on your body weight, on your diet and on your nutritional status. You do not need them only when you eat an unrestricted meal (see below). The tablets may be taken “in the middle of the meal”, at the beginning or at the end, at your choice.	Our diet employs only foods of plant origin (fruit, vegetables, sugars, starches such as pasta and bread, legumes); they must be combined with each other to allow intake of “complete” proteins, with the sufficient quantity of “essential” amino acids, which are those contained in food of animal origin (meat, fish, cheese and eggs) but not in those of vegetable origin (where the proteins are “incomplete” and must be integrated with each other). In order to integrate the proteins, it is important to take them during the day, thus if possible every day at least 2 sources of “starches” (bread, rice, pasta (no egg), semolina (cous- cous, polenta) and at least 1–2 kinds of legumes (peas, chickpeas, beans, broad beans, lentils, etc.), making sure to take at each meal at least one type of starch and one of legumes.
Finally, keep a food diary for a few days between each visit, to allow us scoring quality and quantity of food. If you are asked to bring the 24-h urine collection, please do it in the day preceding the unrestricted meal. ---- (please chose among 1-2-3) UNRESTRICTED MEALS PER WEEK ARE ALLOWED. UNRESTRICTED MEAL IS A MEAL WITHOUT RESTRICTION OF ANY KIND IN QUANTITY AND QUALITY OF THE FOOD. The intake of a sufficient amount of calories is very important, during the day, starting if possible from a “rich” breakfast. It’s important to take a first course, bread, bread sticks, potatoes or other starches. Fruits and vegetables are allowed without restriction, except for fruit in case of diabetes. The dressing (preferably extra virgin olive oil) is to be used without restrictions. Examples of meal distribution:		
BREAKFAST For example biscuits or bread or croissants (all protein-free), jam, tea or coffee, preferably without milk or with soy milk; small amounts of milk are allowed. A lovely cake is fine (with protein free flour). It is important to eat at least a portion of cake, or a few biscuits, or bread, butter and jam, or, for those who do not love the sweet, protein-free bread with oil and tomatoes or other vegetables …	BREAKFAST For example biscuits or bread or croissants, jam, tea or coffee, preferably without milk or with soy milk; in selected cases small amounts of milk are allowed. A lovely cake is fine (a small amount of egg is allowed; however prefer the homemade cakes and egg yolk as compared with egg white). It is important to eat at least a portion of cake, or a few biscuits, or bread, butter and jam, or, for those who do not love the sweet, bread with oil and tomatoes or other vegetables … Take (1-2-3 according to body weight) tablets of alpha-keto analogues	BREAKFAST For example biscuits or bread or croissants, jam, tea or coffee, preferably without milk or with soy milk; small amounts of milk are allowed. A lovely cake is fine; combine different types of flour if you like, It is important to eat at least a portion of cake, or a few biscuits, or bread, butter and jam, or, for those who do not love the sweet, bread (white or raw) with oil and tomatoes or other vegetables …
LUNCH At least one large portion of protein-free pasta or rice with a vegetable based sauce (olive oil without restrictions), with fish, meat or cheese to flavor pasta or rice, at least two servings of vegetables (raw or cooked, without restrictions for quantity and for mode of cooking, including frying, if this is done with olive oil) and at least one fruit after a meal. It also good to associate at least a couple of slices of bread to the vegetables, or breadsticks, rice crackers, etc. Potatoes can be eaten instead of pasta, cooked in any way, but also fried (provided in good olive oil …). Legumes can replace meat, fish, a couple of times per week; better if you use also a little cheese along with legumes (eg pasta and beans with a little parmesan)	LUNCH At least one portion of pasta or rice with a vegetable based sauce (olive oil without restrictions), at least two servings of vegetables (raw or cooked, without restrictions for quantity and for mode of cooking, including frying, if this is done with olive oil) and at least one fruit after a meal. It also good to associate at least a couple of slices of bread to the vegetables, or breadsticks, rice crackers, etc. Potatoes can be eaten instead of the main course, or instead of bread. Take (2-3-4 according to body weight) tablets of alpha-keto analogues	LUNCH At least one large portion of pasta or rice or beans with sauce made of vegetables (extra virgin olive oil without restrictions), at least two servings of vegetables (raw or cooked, with no restrictions even for cooking modes, including fried); if legumes are not employed in addition to the first course, they have to be consume as second course (ie. peas and pasta, or peas with soy beans); at least one fruit after a meal. With vegetables is good associate bread, breadsticks, rice crackers, etc. Potatoes can be eaten instead of the pasta cooked in any way, but also fried (provided in good olive oil …).
DINNER Same as lunch, but the portion of protein free pasta may be smaller. A good alternative is a fine vegetable soup, with vegetables, legumes rice or pasta, bread etc. … and with some meat or cheese. Season with olive oil, providing a good amount of calories. A portion of fish, meat or cheese may be added; else, these aliments may be added to the vegetable portions (spinach with parmesan, egg with spinach, small omelette etc.)	DINNER Same as lunch. A good alternative is a fine vegetable soup, with vegetables, legumes rice or pasta, bread etc. … Season with olive oil, providing a good amount of calories. Take (2-3-4 according to body weight) tablets of alpha-keto analogues	DINNER Same as lunch; a vegetable soup minestrone), with vegetable, potatoes, legumes, rice or pasta, bread may also be a good choice etc. … always season with olive oil, providing good calories. Vegetables can be associated with bread, bread sticks, crackers, rice, etc.
BEVERAGES Drink basically water, but you can include wine (if it is good and not too much…) or beer. Please avoid sodas, since they often have a high amount of added phosphate (not good for the kidney) THIS DIET IS NOT FOR WEIGHT-LOSS The distribution of portions during the day reflects the usual Italian habits (more pasta at noon, less at dinner, but there is no fixed rule). No fear to increase the dose. Careful not to decrease them … and we will control the results together!		
NOTE: for a 0.3 g/Kg/day protein diet: - double the tablets of supplements (1 for each 5Kg of BW) - substitute bread and pasta with protein-free foods and use protein-free flour to prepare biscuits or cakes.		

This diet is based on a series of very simple assumptions: - the first is that the low-protein diet is not only a mean to slow down the progression of kidney failure, but also a very powerful “metabolic regulator”.

- the second is that the proteins of vegetable origin induce a lesser “workload” for the kidney, and therefore, at equal protein content, they are better suited to stabilize the renal function and the metabolism;

- the third is that diets should be followed to improve the quality of life, and hence they have to be followed with some flexibility, and with a certain degree of freedom, progressively adapting to the preferences of each patient: we need your help to identify problems and to solve them together.

- the fourth is that this is almost an “anti-diet” if diet is intended to lose weight: this is a diet rich in pasta, bread and carbohydrates, even if they are different from the usual ones… Sweets and pastry may also be included…

## References

[1] Aparicio M, Bellizzi V, Chauveau P, Cupisti A, Ecder T, Fouque D, Garneata L, Lin S, Mitch WE, Teplan V, Zakar G, Yu X (2012). Ketoacid therapy in predialysis chronic kidney disease patients: final consensus. J Ren Nutr.

[2] Bellizzi V, Cupisti A, Locatelli F (2016). The low-protein diets for chronic kidney disease patients: the Italian experience. BMC Nephrol.

[3] Mitch WE, Remuzzi G (2004). Diets for patients with chronic kidney disease, still worth prescribing. J Am Soc Nephrol.

[4] Levey AS, Greene T, Beck GJ (1999). Dietary protein restriction and the progression of chronic renal disease: what have all of the results of the MDRD study shown? Modification of Diet in Renal Disease Study group. Journal of the American Society of Nephrology: JASN.

[5] Fouque D, Laville M (2009). Low protein diets for chronic kidney disease in non diabetic adults. Cochrane Database Syst Rev.

[6] Thilly N (2013). Low-protein diet in chronic kidney disease: from questions of effectiveness to those of feasibility. Nephrol Dial Transplant.

[7] Kopple JD, Monteon FJ (1986). Effect of energy intake on nitrogen metabolism in non-dialyzed patients with chronic renal failure. Kidney Int.

[8] Laeger T, Henagan TM, Albarado DC, Redman LM, Bray GA, Noland RC, Münzberg H, Hutson SM, Gettys TW, Schwartz MW, Morrison CD (2014). FGF21 is an endocrine signal of protein restriction. J Clin Invest.

[9] D’Alessandro C, Rossi A, Innocenti M, Ricchiuti G, Bozzoli L, Sbragia G, Meola M, Cupisti A (2013). Dietary protein restriction for renal patients: don’t forget protein-free foods. J Ren Nutr.

[10] Ikizler TA, Cano NJ, Franch H, Fouque D, Himmelfarb J, Kalantar-Zadeh K, Kuhlmann MK, Stenvinkel P, TerWee P, Teta D, Wang AY, Wanner C (2013). International Society of Renal Nutrition and Metabolism Prevention and treatment of protein energy wasting in chronic kidney disease patients: a consensus statement by the International Society of Renal Nutrition and Metabolism. Kidney Int.

[11] Isakova T, Wolf MS (2010). FGF23 or PTH: which comes first in CKD?. Kidney Int.

[12] Galassi A, Cupisti A, Santoro A, Cozzolino M (2015). Phosphate balance in ESRD: diet, dialysis and binders against the low evident masked pool. J Nephrol.

[13] Cupisti A, Kalantar-Zadeh K (2013). Management of Natural and Added Dietary Phosphorus Burden in Kidney Disease Seminar. Nephrol.

[14] Benini O, D’Alessandro C, Gianfaldoni D, Cupisti A (2011). Extra-phosphate load from food additives in commonly eaten foods: a real and insidious danger for renal patients. J Ren Nutr.

[15] León JB, Sullivan CM, Sehgal AR (2013). The prevalence of phosphorus-containing food additives in top-selling foods in grocery stores. J Ren Nutr.

[16] D’Alessandro C, Piccoli GB, Cupisti A (2015). The “phosphorus pyramid”: a visual tool for dietary phosphate management in dialysis and CKD patients. BMC Nephrol.

[17] O’Donnell MJ, Yusuf S, Mente A, Gao P, Mann JF, Teo K, McQueen M, Sleight P, Sharma AM, Dans A, Probstfield J, Schmieder RE (2011). Urinary sodium and potassium excretion and risk of cardiovascular events. JAMA.

[18] Tom K, Young VR, Chapman T (1995). Long-term adaptive responses to dietary protein restriction in chronic renal failure. Am J Physiol Endocrinol Metab.

[19] Aparicio M, Bellizzi V, Chauveau P, Cupisti A, Ecder T, Fouque D, Garneata L, Lin S, Mitch W, Teplan V, Yu X, Zakar G (2013). Do Ketoanalogues Still Have a Role in Delaying Dialysis Initiation in CKD Predialysis Patients?. Semin Dial.

[20] Hoseney RC, Rogers DE (1990). The formation and properties of wheat flour doughs. Crit Rev Food Sci Nutr.

[21] Wagner M, Morel MH, Bonicel J, Cuq B (2011). Mechanisms of heat-mediated aggregation of wheat gluten protein upon pasta processing. J Agric Food Chem.

[22] Delcour JA, Joye IJ, Pareyt B, Wilderjans E, Brijs K, Lagrain B (2012). Wheat gluten functionality as a quality determinant in cereal-based food products. Annu Rev Food Sci Technol.

[23] Mauron J (1981). The Maillard reaction in food; a critical review from the nutritional standpoint. Prog Food Nutr Sci.

[24] Sabanis D, Lebesi D, Tzia C (2009). Development of fibre-enriched gluten-free bread: a response surface methodology study. Int J Food Sci Nutr.

[25] Alvarez-Jubete L, Arendt EK, Gallagher E (2009). Nutritive value and chemical composition of pseudocereals as gluten-free ingredients. Int J Food Sci Nutr.

[26] Kang CW, Tungsanga K, Walser M (1986). Effect of the level of dietary protein on the utilization of alpha-ketoisocaproate for protein synthesis. Am J Clin Nutr.

[27] Giordano C (1963). Use of exogenous and endogenous urea for protein synthesis in normal and uremic subjects. J Lab Clin Med.

[28] Giovannetti S, Maggiore Q (1964). A low nitrogen diet with proteins of high biological value for severe chronic uremia. Lancet.

[29] Maroni BJ, Tom K, Masud T, Chapman T, Young VR (1996). How is lean body mass conserved with the very-low protein diet regimen?. Miner Electrolyte Metab.

[30] Mitch WE, Walser M, Sapir DG (1981). Nitrogen sparing induced by leucine compared with that induced by its keto analogue, alpha-ketoisocaproato, in fasting obese man. J Clin Invest.

[31] Wang DT, Lu L, Shi Y (2014). Supplementation of ketoacids contributes to the up regulation of the Wnt7a/Akt/p70S6K pathway and the down-regulation of apoptotic and ubiquitin-proteasome system in the muscle of 5/6 nephrectomized rats. Br J Nutr.

[32] Feiten SF, Draibe SA, Watanabe R (2005). Short-term effects of a very low protein diet supplemented with ketoacids in non-dialyzed chronic kidney disease patients. Eur J Clin Nutr.

[33] Goraya N, Wesson DE (2012). Acid–base status and progression of chronic kidney disease. Curr Opin Nephrol Hypertens.

[34] Malvy D, Maingourd C, Pengloan J (1999). Effects of severe protein restriction with ketoanalogues in advanced renal failure. J Am Coll Nutr.

[35] Barsotti G, Morelli E, Cupisti A, Meola M, Dani L, Giovannetti S (1996). A low-nitrogen low-phosphorus vegan diet for patients with chronic renal failure. Nephron.

[36] Cupisti A, Morelli E, Meola M, Barsotti M, Barsotti G (2002). Vegetarian diet alternated with conventional low-protein diet for patients with chronic renal failure. J Renal Nutr.

[37] Bellizzi V (2013). Low-protein diet or nutritional therapy in chronic kidney disease?. Blood Purif.

[38] Shah AP, Kalantar-Zadeh K, Kopple JD (2015). Is there a role for ketoacid supplements in the management of CKD?. Am J Kidney Dis.

[39] Milas NC, Nowalk MP, Akpele L, Castaldo L, Coyne T, Doroshenko L, Kigawa L, Korzec-Ramirez D, Scherch LK, Snetselaar L (1995). Factors associated with adherence to the dietary protein intervention in the Modification of Diet in Renal Disease Study. J Am Diet Assoc.

[40] Paes-Barreto JG, Silva MI, Qureshi AR, Bregman R, Cervante VF, Carrero JJ, Avesani CM (2013). Can renal nutrition education improve adherence to a low-protein diet in patients with stages 3 to 5 chronic kidney disease?. J Ren Nutr.

[41] Dolecek TA, Olson MB, Caggiula AW (1995). Registered dietitian time requirements in the Modificaton of Diet in Renal Disease Study. J Am Diet Assoc.

[42] Bellizzi V, Di Iorio BR, Brunori G, De Nicola L, Minutolo R, Conte G, Cianciaruso B, Scalfi L (2010). Assessment of nutritional practice in Italian chronic kidney disease clinics: a questionnaire-based survey. J Ren Nutr.

[43] Kopple JD, Berg R, Houser H, Steinman TI, Teschan P (1989). Nutritional status of patients with different levels of chronic renal insufficiency. Modification of Diet in Renal Disease (MDRD) Study Group. Kidney Int.

[44] De Nicola L, Minutolo R, Bellizzi V, Zoccali C, Cianciaruso B, Andreucci VE, Fuiano G, Conte G, for the investigators of the TArget Blood Pressure LEvels in Chronic Kidney Disease (TABLE in CKD) Study Group (2004). Achievement of Target Blood Pressure Levels in Chronic Kidney Disease: A Salty Question?. Am J Kidney Dis.

[45] Pisani A, Riccio E, Bellizzi V, Caputo DL, Mozzillo G, Amato M, Andreucci M, Cianciaruso B, Sabbatini M (2016). 6-tips diet: a simplified dietary approach in patients with chronic renal disease. A clinical randomized trial. Clin Exp Nephrol.

[46] Piccoli GB, Deagostini MC, Vigotti FN, Ferraresi M, Moro I, Consiglio V, Scognamiglio S, Mongilardi E, Clari R, Aroasio E, Versino E, Porpiglia F (2014). Which low-protein diet for which CKD patient? An observational, personalized approach. Nutrition.

[47] Maroni BJ, Steinman TI, Mitch WE (1985). A method for estimating nitrogen intake of patients with chronic renal failure. Kidney Int.

[48] De Nicola L, Minutolo R, Chiodini P, TArget Blood Pressure LEvels in Chronic Kidney Disease (TABLE in CKD) Study Group (2006). Global approach to cardiovascular risk in chronic kidney disease: reality and opportunities for intervention. Kidney Int.

[49] Cupisti A, Bottai A, Bellizzi V, Brunori G, Cianciaruso B, De Nicola L, Oldrizzi L, Quintaliani G, Santoro D, Di Iorio BR (2015). Characteristics of patients with chronic kidney disease referred to a nephrology outpatient clinic: results of Nefrodata study. G Ital Nefrol.

[50] Locatelli F, Del Vecchio L (2014). Protein restriction: a revisited old strategy with new opportunities?. Nephrol Dial Transplant.

